# Inhibition of dorsal raphe GABAergic neurons blocks hyperalgesia during heroin withdrawal

**DOI:** 10.1038/s41386-023-01620-5

**Published:** 2023-06-03

**Authors:** Yocasta Alvarez-Bagnarol, Raul García, Leandro F. Vendruscolo, Marisela Morales

**Affiliations:** 1 Neuronal Networks Section, Integrative Neuroscience Research Branch, National Institute on Drug Abuse, Intramural Research Program, Baltimore, MD USA; 2 Department of Anatomy and Neurobiology, University of Puerto Rico, Medical Sciences Campus, San Juan, Puerto Rico; 3 Stress and Addiction Neuroscience Unit, Integrative Neuroscience Research Branch, National Institute on Drug Abuse and National Institute on Alcohol Abuse and Alcoholism, Intramural Research Programs, Baltimore, MD USA

**Keywords:** Cellular neuroscience, Motivation

## Abstract

Opioid withdrawal signs, such as hyperalgesia, are manifestations of opioid use disorder that may contribute to opioid seeking and taking. We have previously identified an association between dorsal raphe (DR) neurons and the expression of hyperalgesia during spontaneous heroin withdrawal. Here, we found that chemogenetic inhibition of DR neurons decreased hyperalgesia during spontaneous heroin withdrawal in male and female C57/B6 mice. By neuroanatomy, we identified three major subtypes of DR neurons expressing μ-opioid receptors (MOR) that were activated in hyperalgesia during spontaneous withdrawal, those expressing vesicular GABA transporter (VGaT), glutamate transporter 3 (VGluT3), or co-expressing VGluT3 and tryptophan hydroxylase (TPH). In contrast, we identified a small population of DR-MOR neurons expressing solely TPH, which were not activated in hyperalgesia during spontaneous withdrawal. Collectively, these findings indicate a role of the DR in hyperalgesia during spontaneous heroin withdrawal mediated, in part, by the activation of local MOR-GABAergic, MOR-glutamatergic and MOR-co-releasing glutamatergic-serotonergic neurons. We found that  specific chemogenetic inhibition of DR-VGaT neurons blocked hyperalgesia during spontaneous heroin withdrawal in male and female mice. Collectively, these findings indicate that DR-GABAergic neurons play a role in the expression of hyperalgesia during spontaneous heroin withdrawal.

## Introduction

The number of opioid-related fatalities continues to rise in the United States [1]. The poor rates of compliance and low adherence to treatment for opioid use disorder (OUD) suggest that new approaches for prevention, diagnosis and treatment are needed [2]. Investigating neuroadaptations that occur in brain regions and cell types in opioid dependence will improve our understanding of the etiology of OUD.

Negative emotional states that emerge during opioid withdrawal are hypothesized to drive compulsive drug-taking behavior in patients with OUD [3, 4]. Somatic (“physical”) and motivational symptoms of opioid withdrawal include diarrhea, insomnia, fever, pain, dysphoria, anxiety, and depression. Hyperalgesia, defined as sensitization to noxious and painful stimuli, is a long-lasting symptom of opioid withdrawal [5] that is hypothesized to contribute to the continued drug use to alleviate the pain via negative reinforcement [6]. In patients with OUD, cue-induced craving has been associated with an increase in pain sensitivity after several months of abstinence [7]. In laboratory animals, hyperalgesia has been reported in fentanyl-dependent mice that exhibited drug-intake escalation [8]. We recently identified associations of dorsal raphe (DR) increases of c-Fos cellular expression with hyperalgesia during spontaneous heroin withdrawal in male and female heroin-dependent mice [9]. These findings indicate a possible role of DR in hyperalgesia in opioid dependence.

Pharmacological studies have shown changes in the DR serotonergic system associated with opioids. For example, studies in the DR of morphine-dependent rodents have found increases in serotonin (5-hydroxytryptamine, [5-HT]) turnover after spontaneous withdrawal [10], and decreases in extracellular levels of 5-HT after naltrexone precipitated withdrawal [11]. In regards to serotonergic receptors, a decrease in functional coupling of 5-HT1A receptor to G-proteins was found in the DR of morphine-dependent mice after spontaneous withdrawal [12]. In addition to changes in 5-HT levels and serotonergic receptor function, electrophysiological recordings in morphine-dependent rats detected a higher frequency of spontaneous γ-aminobutyric acid (GABA)ergic inhibitory postsynaptic currents in DR 5-HT neurons during withdrawal [13]. These studies suggest that opioid withdrawal modulates DR-serotonergic and -GABAergic systems.

While the DR is best known for containing serotonergic neurons [14, 15], the DR is heterogenous in its cellular composition, which includes peptidergic, GABAergic, glutamatergic, and dopaminergic neurons organized in different subdivisions of the DR [16–18]. In addition, the expression of the µ-opioid receptor (MOR), which is the primary mediator of opioid’s effects [19], has been detected in neurons distributed in the caudal regions of the rodent DR [20, 21]. Moreover, DR-targeted gene knockout of MORs in heroin-dependent mice prevented the reduction of social preference during spontaneous withdrawal, demonstrating a role of DR-MORs in opioid withdrawal [22]. However, it is unclear the extent to which MORs are expressed in specific subtypes of DR neurons, such as vesicular GABA transporter (VGaT), vesicular glutamate transporter 3 (VGluT3), tryptophan hydroxylase (TPH), or their combinations, and whether MOR present in specific types of DR-neurons mediate opioid withdrawal effects.

Here, we examined the effect of global inhibition of DR neurons in hyperalgesia during spontaneous heroin withdrawal, and given that heroin activates MOR, we then phenotyped subpopulations of DR neurons expressing MOR to determine their possible involvement in hyperalgesia. By combination of phenotyping of DR-MOR and co-expression of c-Fos protein as a maker of neuronal activation, we determined the extent to which hyperalgesia activated specific subtypes of DR neurons. We found that DR-GABAergic neurons expressing MOR were one of the neuronal subpopulations that were activated during hyperalgesia. Next, we determined the extent to which DR-GABAergic neurons play a role in hyperalgesia by selective chemogenetic inhibition of DR-GABAergic neurons during spontaneous heroin withdrawal.

## Methods

Methodological details and resources table (Table S1) are described in supplementary material.

### Animals

We used 8-week-old male and female wild-type C57/B6  mice for the behavioral and anatomical studies. We used male and female VGaT::Cre mice (Slc32a1, in C57/B6  background from The Jackson Laboratories, Bah Harbor, ME), bred in the NIDA/IRP animal facility, for behavioral studies. All mice were group-housed (three per cage) based on sex and treatment condition in a temperature-controlled (23 °C) vivarium on a 12/12 h light/dark cycle (lights on at 7 AM). The mice had *ad libitum* access to food and water except during the experimental sessions. The study adhered to the National Institutes of Health Guide for the Care and Use of Laboratory Animals and approved by the National Institute on Drug Abuse Animal Care and Use Committee.

### Surgery

For wild-type mice, we used an adeno-associated virus (AAV, serotype 2) encoding hM4Di (inhibitory Gi-coupled Designer Receptor Exclusively Activated by Designer Drugs [DREADDs]) tethered to the red fluorescent protein (mCherry) under the regulation of hSyn promoter. For VGaT::Cre mice, we used either the Cre-dependent AAV2 encoding hM4Di tethered to mCherry, or mCherry under the regulation of hSyn promoter. We injected 200 nl of AAV2-hSyn-hM4Di-mCherry, AAV2-hSyn-DIO-hM4Di-mCherry or AAV2-hSyn-DIO-mCherry in the DR at the following stereotaxic coordinates: anterior/posterior, -4.2; medial/lateral, 0; dorsal ventral, -3.2 mm, from bregma [23].

### Chemogenetic inhibition of the DR in hyperalgesia during spontaneous heroin withdrawal

Six weeks after the viral injections, we collected baseline measurements of hyperalgesia by evaluating mechanical sensitivity using an electronic von Frey device (Ugo Basile, VA, Italy). We habituated wild-type mice (11 males, 10 females) or the VGaT::Cre mice (16 males, 21 females) to the testing room for at least 30 min. The mice were then placed in the testing apparatus for at least 1 h and began testing when exploratory behaviors ceased. Using a von Frey device, we obtained the average of the six measurements of paw withdrawal thresholds for each mouse and considered it a measure of mechanical sensitivity. Next, we assigned the mice to the experimental groups, matched by baseline for mechanical sensitivity. One group received repeated injections of saline (10 ml/kg, s.c.), and the other group received increasing doses of heroin (5, 10, 20, and 40 mg/kg, s.c.) twice daily (7 AM and 7 PM) for four consecutive days.

**Fig. 1:**
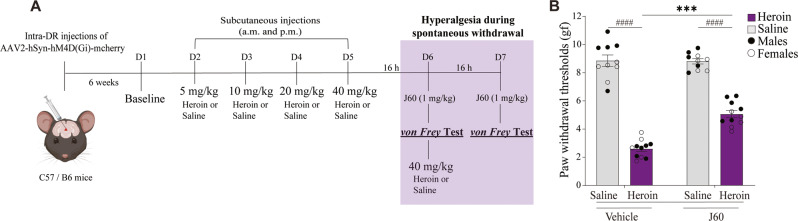
Chemogenetic inhibition of DR neurons decreases hyperalgesia during heroin withdrawal. **A** Timeline and experimental procedures. Adult male and female C57/B6 mice received injections of AAV2-hM4Di-mCherry in the DR. Measurement of baseline paw withdrawal thresholds on day 1 (D1). Twice daily subcutaneous injections of saline (10 ml/kg; nondependent) or increasing doses of heroin (5–40 mg/kg; dependent) from D2 to D5. Paw withdrawal thresholds test 16 h after the last heroin injection on D6 and D7. For DR chemogenetic inhibition, mice received intraperitoneal (i.p.) of vehicle (saline; 10 ml/kg) or J60 (1 mg/kg) 30 min before hyperalgesia testing (D6 and D7). **B** Heroin-treated mice exhibited lower paw withdrawal thresholds (hyperalgesia) than saline-treated mice (^####^*p* < 0.0001). Thresholds were increased by i.p. injections of J60 (***p = 0.0003). *n* = 10–11/group. gf, grams of force. The data are expressed as mean ± SEM.

Approximately 16 h after the last saline or heroin injection, the mice were injected with vehicle (saline, 10 ml/kg, i.p.) or J60 (1 mg/kg, i.p.) 30 min before assessing paw withdrawal thresholds (Fig. 1A). The dose and pretreatment time were based on previously published work [24]. The J60 injections were counterbalanced across experimental days, with half of the mice receiving vehicle/J60 and the other half receiving J60/vehicle on any given day. In the evening of the same day of von Frey testing, the mice were injected with saline or heroin (40 mg/kg, s.c.). The next day, approximately 16 h after the saline or heroin injection, we repeated the same experimental procedures as the prior day with counterbalanced vehicle and J60 injections.

### RNAscope in situ hybridization combined with immunohistochemistry

We perfused mice 1 h after the end of von Frey testing and collected coronal serial cryosections (16 μm thick) from the DR of heroin and saline-treated mice. We selected heroin and saline-treated mice that exhibited robust expression of hyperalgesia to detect c-Fos protein expression. To reduce c-Fos staining variability that can be caused by extraneous factors, we performed parallel immunohistological staining of a set of sections from mice of each treatment group. For the immunodetection of c-Fos and TPH, free-floating coronal sections were incubated for 2 h at 30 °C with rabbit anti-phospho-c-Fos (1:200) and mouse anti-TPH (1:1000) in DEPC-treated PB with 4% BSA, 0.3% Triton X-100, and RNasin (5 μl/1 ml). After rinsing three times (10 min each) with DEPC-treated PB, the sections were incubated with donkey anti-rabbit Alexa Fluor-647 (1:100) and donkey anti-mouse Alexa Fluor-750 (1:100) secondary antibodies for 1 h at 30 °C. The sections were rinsed with DEPC-treated PB, mounted and dried overnight. In situ hybridization was performed using the RNAscope Multiplex Fluorescent v1 assay according to the manufacturer’s instructions. The sections were treated with heat and protease digestion followed by hybridization with a mixture containing target probes to mouse VGaT or VGluT3 and *Oprm1*. *Oprm1* was detected by Atto-550, and VGaT or VGluT3 were detected by Alexa Fluor-488.

### Statistical analysis

The number of mice that were used in each group or condition is described in each figure legend. We analyzed paw withdrawal thresholds for wild-type mice using two-way repeated-measures analysis of variance (ANOVA) with group (saline *vs*. heroin) as a between-subjects factor and chemogenetic treatment (vehicle *vs*. J60) as a within-subjects factor. We analyzed paw withdrawal thresholds for VGaT::Cre mice using three-way repeated-measures ANOVA, with viral vector (hM4Di-mCherry *vs*. mCherry) and group as a between-subjects factor and chemogenetic treatment as a within-subjects factor. *Post hoc* multiple comparisons with Sidak correction were performed. Mice with virus injections outside the targeted structure were excluded from the analysis (*n* = 4 saline; *n* = 2 heroin). We analyzed anatomical data using Student’s *t* test to compare differences between groups and one-way repeated-measures ANOVA for comparisons among DR subregions.

## Results

### Inhibition of DR neurons decreases hyperalgesia

We have identified an association between the activity of DR neurons and the expression of hyperalgesia during heroin withdrawal [9]. To determine the role of DR in heroin withdrawal, we studied hyperalgesia in mice expressing hM4Di in DR neurons by injecting an adeno-associated virus vector encoding hM4Di in the DR of mice (Fig. S1). To examine the effects of chemogenetic inhibition of hM4Di-expressing DR neurons during hyperalgesia testing, mice were injected with an hM4Di ligand J60 (1 mg/kg, i.p.) 30 min before the assessment of mechanical sensitivity at 16 h of withdrawal (Fig. 1A). Using a two-way ANOVA, we identified significant effects of group (*F*_1,19_ = 383, *p* < 0.0001) and chemogenetic treatment (*F*_1,19_ = 18.69, *p* = 0.0004) and a significant group × chemogenetic treatment interaction (*F*_1,19_ = 19.87, *p* = 0.0003; Fig. 1B) on paw withdrawal thresholds. By *post hoc* comparisons, we found that heroin-treated mice exhibited lower paw withdrawal thresholds (hyperalgesia) than saline-treated mice (*p* < 0.0001). Treatment with J60 increased paw withdrawal thresholds (attenuated hyperalgesia) in heroin-treated mice compared with vehicle (*p* < 0.0001).

### Subtypes of DR neurons expressing MOR mRNA in drug-naïve mice

**Fig. 2:**
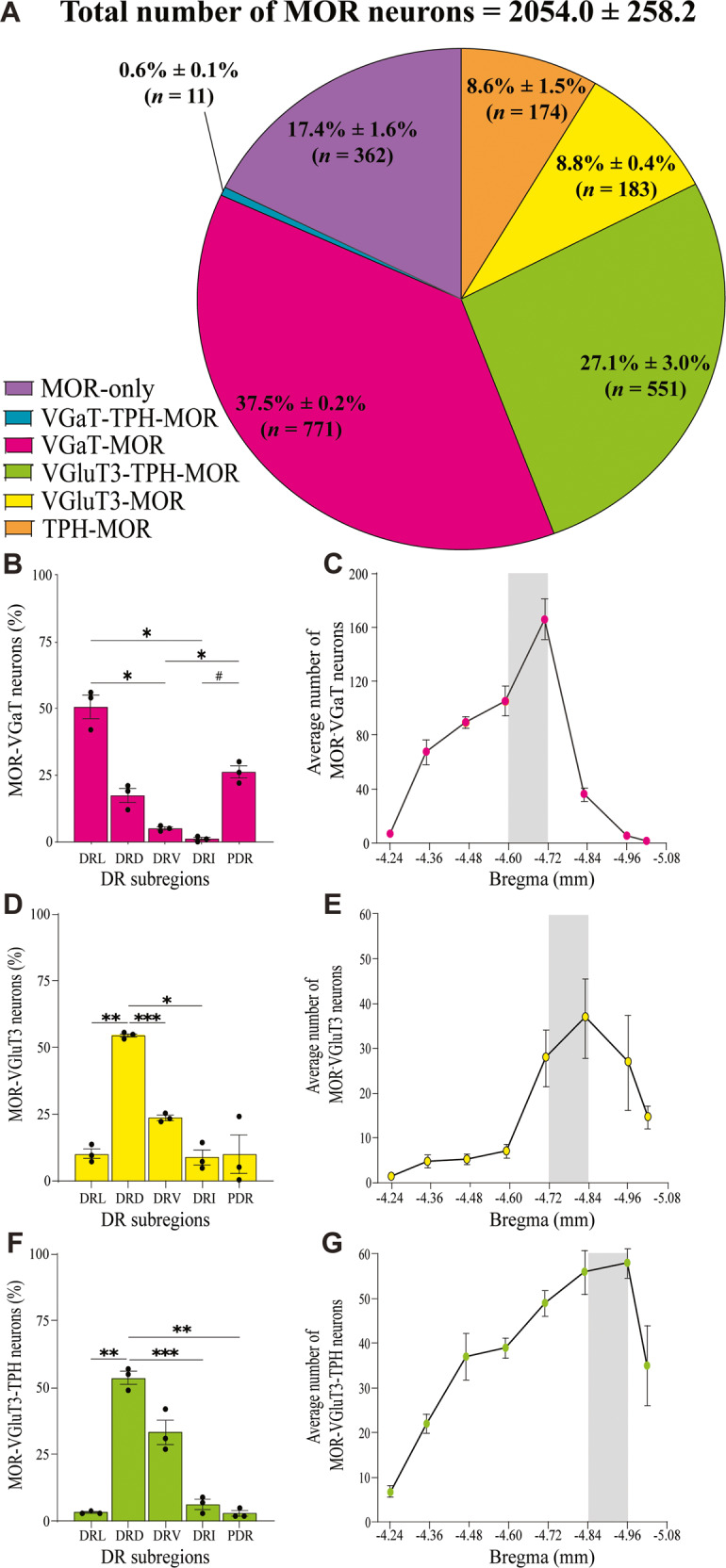
Distribution of subpopulations of DR neurons expressing MOR in drug-naïve mice. **A** Proportion of subpopulations of DR neurons expressing MOR mRNA. **B**, **C** DR topographic distribution of MOR-VGaT neurons. **B** MOR-VGaT neurons were more concentrated in DR lateral subregion (DRL) than DR ventromedial (DRV; *p = 0.0346) and DR interfascicular (DRI; *p = 0.0230) subregions; and more concentrated in the DR posterodorsal (PDR) than the DRV and DRI (*p = 0.0296, ^#^*p* = 0.0210) subregions. **C** DR rostrocaudal distribution of MOR-VGaT neurons. **D**, **E** DR topographic distribution of MOR-VGluT3 neurons. **D** MOR-VGluT3neurons were more concentrated in DR dorsomedial (DRD) subregion than in DRL (**p = 0.0042), DRV (***p = 0.0005) and DRI (*p = 0.0186) subregions. **E** DR rostrocaudal distribution of MOR-VGluT3 neurons. **F–G** DR topographic distribution of MOR-VGluT3-TPH neurons. **F** MOR-VGluT3-TPH neurons were more concentrated in DRD subregion than in the DRL (**p = 0.0095), DRI (***p = 0.0010) and PDR (**p = 0.0070) subregions. **G** DR rostrocaudal distribution of MOR-VGluT3-TPH neurons. The data are expressed as mean ± SEM (*n* = 3 mice/group).

To identify the subpopulations of DR neurons that express MOR mRNA, we combined immunohistochemistry (for the detection of TPH) and RNAscope in situ hybridization (for the detection of transcripts encoding MOR, VGaT or VGluT3) (Fig. 2). We found that within the total population of DR neurons expressing MOR mRNA (2054 ± 258.2 neurons, three mice; Fig. 2A), 17.4% ± 1.6% (362/2054) expressed MOR mRNA alone without serotonergic, glutamatergic or GABAergic markers. In contrast, most MOR mRNA expressing neurons co-expressed GABAergic (VGaT) or glutamatergic (VGluT3) markers: 37.5% ± 0.2% co-expressed MOR mRNA and VGaT mRNA (771/2054), 27.1% ± 3.0% co-expressed MOR mRNA, VGluT3 mRNA and TPH protein (551/2054), 8.8% ± 0.4% co-expressed MOR mRNA and VGluT3 mRNA (183/2054), 8.6% ± 1.5% co-expressed MOR mRNA and TPH protein (174/2054) and, as little as 0.6% ± 0.1% co-expressed MOR mRNA, VGaT mRNA and TPH protein (11/2054).

Using one-way repeated-measure ANOVAs, we found different percentages of MOR-VGaT (*F*_1.382,2.763_ = 48.21, *p* = 0.0075; Fig. 2B), MOR-VGluT3 (*F*_1.150,2.301_ = 27.54, *p* = 0.0252; Fig. 2D), and MOR-VGluT3-TPH (*F*_1.122,2.245_ = 68.62, *p* = 0.0100; Fig. 2F) neurons in specific DR subregions. By *post hoc* comparisons, we found higher percentage of MOR-VGaT neurons in the DR lateral (DRL) than in the ventral (DRV) (*p* = 0.0346) and interfascicular (DRI) (*p* = 0.0230) subregions; and in the DR posterodorsal (PDR) than in the DRV and DRI (*p* = 0.0296, *p* = 0.0210) subregion. Within the DR rostral-caudal levels, we detected the highest concentration of MOR-VGaT neurons in the middle portion of the DR (–4.72 mm; Fig. 2C). In contrast to the distribution of DR-MOR-VGaT neurons, we detected higher percentage of MOR-VGluT3 neurons in the DRD subregion than in the DRL (*p* = 0.0042), DRV (*p* = 0.0005) and DRI (*p* = 0.0186) subregions. In common with the distribution of MOR-VGluT3 neurons, we observed higher percentage of MOR-VGluT3-TPH neurons in the DRD subregion than in the DRL (*p* = 0.0095), DRI (*p* = 0.0010) and PDR (*p* = 0.0070) subregions. Within the DR rostral-caudal levels, we found the highest concentration of MOR-VGluT3 (-4.84 mm) and MOR-VGluT3-TPH neurons (-4.96 mm) in the caudal portion of the DR. (Fig. 2E, G).

### c-Fos expression in DR MOR-VGaT neurons in hyperalgesia

**Fig. 3:**
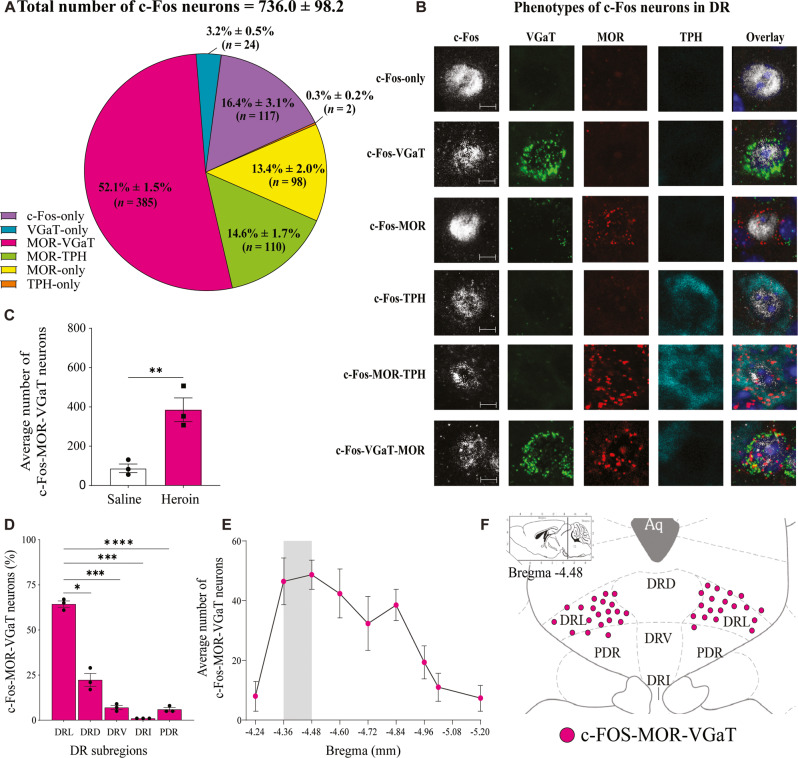
DR expression of c-Fos in MOR-VGaT neurons in hyperalgesia during spontaneous heroin withdrawal. **A** Proportion of subpopulations of DR neurons expressing c-Fos. **B** DR confocal images of the subpopulation of neurons expressing c-Fos at a magnification of 20X, showing detection of c-Fos protein (white), VGaT mRNA (green), MOR mRNA (red), or TPH protein (cyan). **C** Heroin-treated mice exhibited a higher number of c-Fos-MOR-VGaT neurons than saline-treated mice (**p = 0.0048). **D** DR regional distribution of c-Fos-MOR-VGAT neurons in heroin treated mice showing higher concentration in the DR lateral subregion (DRL) than in the DR dorsomedial (DRD; *p = 0.0155), DR ventromedial (DRV; ***p = 0.0005), DR interfascicular (DRI; ***p = 0.0008), or DR posterodorsal (PDR; ****p = 0.0004) subregions. **E** DR rostrocaudal distribution of c-Fos-MOR-VGAT neurons. **F** DR schematic representation showing concentration of c-Fos-MOR-VGAT neurons in the DRL subregion at bregma -4.48. The data in (**A**, **C**, **D** and **E**) are expressed as mean ± SEM. (*n* = 3 mice/group). Aq aqueduct. Scale bars: 5 µm (**B**).

To identify the subtypes of activated DR neurons following the assessment of hyperalgesia, we detected neuronal activation within the DR by immunodetection of c-Fos and found that heroin-treated mice exhibited a higher number of c-Fos-expressing neurons than saline-treated mice (Student’s *t* test, *t*_4_ = 4.983, *p* = 0.0038; Fig. S2B, C). Next, we phenotyped DR c-Fos expressing neurons in heroin- and saline-treated mice by immunodetection of TPH, and RNAscope in situ hybridization for detection of transcripts encoding MOR or VGaT (Fig. 3; Fig. S2A and Fig. S3). We found that 20.0% ± 2.6% of the total population of c-Fos-expressing DR neurons in heroin-treated mice (736.0 ± 98.2 neurons, three mice; Fig. 3A) lacked MOR mRNA: 16.4% ± 3.1% expressed c-Fos protein (117/736), 3.2% ± 0.5% expressed VGaT mRNA (24/736), and 0.3% ± 0.2% expressed TPH protein (2/736). In contrast, 80.0% ± 2.6% of c-Fos-expressing DR neurons co-expressed MOR mRNA: 52.1% ± 1.5% co-expressed MOR mRNA and VGaT mRNA (385/736; Fig. S4), 14.6% ± 1.7% co-expressed MOR mRNA and TPH protein (110/736) and 13.4% ± 2.0% co-expressed MOR mRNA alone (98/736).

We further confirmed that heroin-treated mice exhibited a higher number of c-Fos-MOR-VGaT neurons than saline-treated mice (Student’s *t* test, *t*_4_ = 4.653, *p* = 0.0048; Fig. 3C), and by one-way ANOVA, we found different percentages of c-Fos-MOR-VGaT neurons in specific DR subregions (*F*_1.141,2.281_ = 150.9, *p* = 0.0039). By *post hoc* comparisons, we found higher percentage of c-Fos-MOR-VGaT neurons in the DRL than in the DRD (*p* = 0.0155), DRV (*p* = 0.0005), DRI (*p* = 0.0008), and PDR (*p* = 0.0004; Fig. 3D) subregions. Within the DR rostral-caudal levels, we found the highest concentration of c-Fos-MOR-VGaT neurons in the rostral aspect of the DR (-4.48 mm; Fig. 3E). Distribution of subpopulations in the DR of c-Fos neurons lacking MOR mRNA are shown in Figure S5.

### c-Fos expression in DR MOR-VGluT3 and MOR-VGluT3-TPH neurons in hyperalgesia

**Fig. 4:**
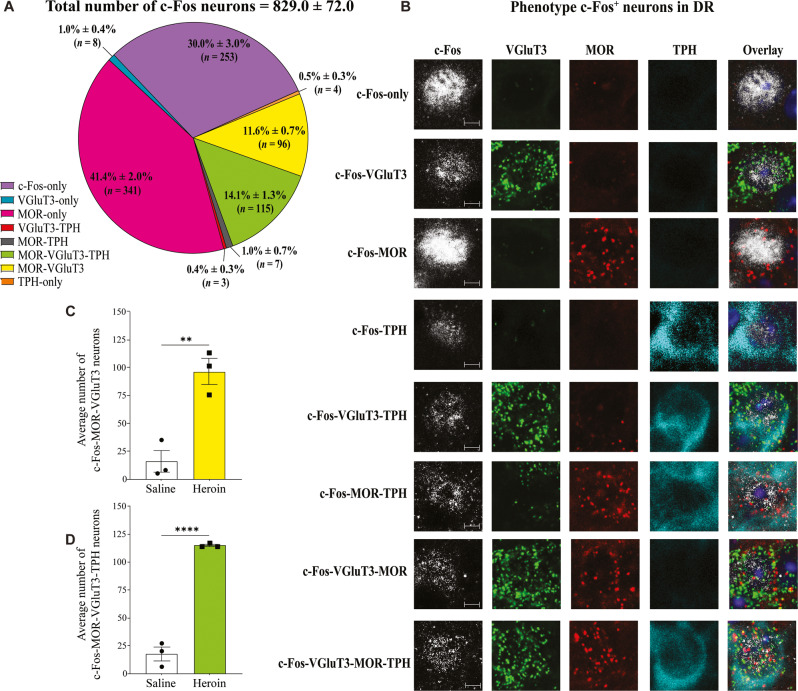
DR expression of c-Fos in MOR-VGluT3 and MOR-VGluT3-TPH neurons in hyperalgesia during spontaneous heroin withdrawal. **A** Proportion of subpopulations of DR neurons expressing c-Fos. **B** DR confocal images of the subpopulation of neurons expressing c-Fos at a magnification of ×20, showing detection of c-Fos protein (white), VGluT3 mRNA (green), MOR mRNA (red), or TPH protein (cyan). **C** Heroin-treated mice exhibited a higher number of c-Fos-MOR-VGluT3 neurons than saline-treated mice (**p = 0.0027). **D** Heroin-treated mice exhibited a higher number of c-Fos-MOR-VGluT3-TPH neurons than saline-treated mice (**p < 0.0001). The data in (**A**, **C** and **D**) are expressed as mean ± SEM. (*n* = 3 mice/group). Scale bars: 5 µm (**B**).

We had previously demonstrated that two types of DR-VGluT3 (VGluT3-only and dual VGluT3-TPH) neurons are involved in motivated behavior [25, 26]. Therefore, we next evaluated the possible involvement of DR-VGluT3 neurons in hyperalgesia by conducting a second series of experiments in tissue from the same mice that were used for the DR MOR-VGaT analysis. After confirming a higher number of c-Fos-expressing in heroin-treated mice than saline-treated mice (Student’s *t* test, *t*_4_ = 9.363, *p* = 0.0004; Fig. S6B, C), we phenotyped DR c-Fos expressing neurons in heroin and saline-treated mice by immunodetection of TPH, and dual RNAscope in situ hybridization for detection of transcripts encoding MOR or VGluT3 (Fig. 4; Fig. S6A and Fig. S7). We found that 32.0% ± 3.0% of the total population of c-Fos-expressing DR neurons in heroin-treated mice (829.0 ± 72.0 neurons, three mice; Fig. 4A) lacked MOR mRNA: 30.0% ± 3.0% expressed c-Fos protein (253/829), 1.0% ± 0.4% expressed VGluT3 mRNA (8/829), 0.5% ± 0.3% expressed TPH protein (4/829), and 0.4% ± 0.3% expressed VGluT3 mRNA and TPH protein (3/829). In contrast, 68.0% ± 3.0% of c-Fos-expressing DR neurons co-expressed MOR mRNA: 41.4% ± 2.0% expressed MOR mRNA (341/829), 14.1% ± 1.3% co-expressed MOR mRNA, VGluT3 mRNA, and TPH protein (115/829; Fig. S8), 11.6% ± 0.7% co-expressed MOR mRNA and VGluT3 mRNA (96/829), and 1.0% ± 0.7% expressed MOR mRNA and TPH protein (7/829).

We further confirmed that heroin-treated mice exhibited a higher number of c-Fos-MOR-VGluT3 (Student’s *t* test, *t*_4_ = 5.456, *p* = 0.0027; Fig. 4C) and c-Fos-MOR-VGluT3-TPH neurons (*t*_4_ = 15.56, *p* < 0.0001; Fig. 4D) than saline-treated mice, and by one-way ANOVA, we found different percentages of c-Fos-MOR-VGluT3 neurons (*F*_1.496,2.992_ = 21.11, *p* = 0.0181) and c-Fos-MOR-VGluT3-TPH neurons (*F*_1.070,2.140_ = 52.86, *p* = 0.0152) in specific DR subregions. By *post hoc* comparisons, we found higher percentage of c-Fos-MOR-VGluT3 neurons in the DRD than in the DRL (*p* = 0.0367), DRI (*p* = 0.0163), and PDR (*p* = 0.0105) subregions and in the DRV than in the PDR subregion (*p* = 0.0087; Fig. S9A). We also found higher percentage of c-Fos-MOR-VGluT3-TPH neurons in the DRD than in the DRL (*p* = 0.0074), DRI (*p* = 0.0077), and PDR (*p* = 0.0066) subregions and in the DRV than in the DRL (*p* = 0.0225) and PDR (*p* = 0.0237; Fig. S9C) subregions. Within the DR rostral-caudal levels, we found the highest concentration of c-Fos-MOR-VGluT3 and c-Fos-MOR-VGluT3-TPH neurons in the caudal portion of the DR (-4.84 mm; Fig. S9B, D). Distribution of subpopulations in the DR of c-Fos neurons lacking MOR mRNA are shown in Fig. S10.

### Inhibition of DR-VGaT neurons blocks hyperalgesia

**Fig. 5:**
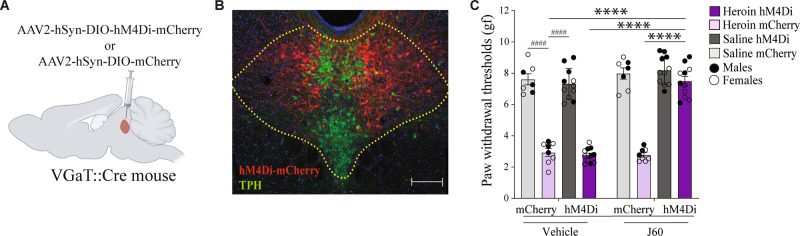
Inhibition of DR-VGaT neurons blocks hyperalgesia during heroin withdrawal. **A** DR viral injections of AAV2-hSyn-DIO-hM4Di-mCherry or AAV2-hSyn-DIO-mCherry in VGaT::Cre mice (*n* = 37). **B** Representative image (magnification of ×5) of the expression of AAV2-DIO-hSyn-hM4D (Gi)-mCherry in DR-VGaT neurons at bregma -4.36 mm from a heroin-treated VGaT::Cre mouse. **C** Heroin-treated mCherry control mice exhibited lower paw withdrawal thresholds (hyperalgesia) than saline-treated hM4Di-mCherry (^####^*p* < 0.0001) and mCherry control mice (^####^ *p* < 0.0001). I.p. injections of J60 increased thresholds in heroin-treated hM4Di-mCherry mice compared with vehicle (****p < 0.0001) and with heroin-treated mCherry control mice treated with either vehicle (****p < 0.0001) or J60 (****p < 0.0001). *n* = 16-21/group. gf, grams of force. The data in (**C**) are expressed as mean ± SEM. Scale bars: 100 µm (**B**).

Given that 52.1% ± 1.5% of DR-c-Fos-expressing neurons co-expressed MOR mRNA and VGaT mRNA (Fig. 3A), we next used a chemogenetic approach to selectively inhibit DR-VGaT neurons and tested their role in hyperalgesia during spontaneous withdrawal. For these studies we did intra-DR injection in VGaT::Cre mice of a Cre-dependent AAV2 encoding hM4Di tethered to mCherry (AAV2-hSyn-DIO-hM4Di-mCherry; hM4Di-mCherry mice; Fig. 5A, B), or the control viral vector (AAV2-hSyn-DIO-mCherry; mCherry control mice) and injected them with saline (vehicle) or the ligand, J60 (1 mg/kg, i.p.), 30 min before the assessment of mechanical sensitivity at 16 h of spontaneous withdrawal. Using a three-way ANOVA, we identified significant effects of group (*F*_1,33_ = 274.3, *p* < 0.0001), chemogenetic treatment (*F*_1,33_ = 84.87, *p* < 0.0001) and viral vector (*F*_1,33_ = 23.75, *p* < 0.0001), and group × chemogenetic treatment interaction × viral vector interaction (*F*_1,33_ = 48.68, *p* < 0.0001; Fig. 5C) on paw withdrawal thresholds. By *post hoc* comparisons, we found that heroin-treated mCherry control mice exhibited lower paw withdrawal thresholds (hyperalgesia) than saline-treated hM4Di-mCherry (*p* < 0.0001) or mCherry control mice (*p* < 0.0001). Mice injected with J60 showed increased paw withdrawal thresholds (attenuated hyperalgesia) in heroin-treated hM4Di-mCherry mice compared with mice injected with vehicle (*p* < 0.0001) or with heroin-treated mCherry control mice injected with either J60 (*p* < 0.0001) or vehicle (*p* < 0.0001). These chemogenetic results indicate that DR-VGaT neurons mediate the expression of hyperalgesia during heroin withdrawal.

## Discussion

We had demonstrated that the activity of DR neurons, measured by the detection of c-Fos expression, was associated with hyperalgesia in spontaneous heroin withdrawal [9]. We now demonstrated that non-specific inhibition of DR-neurons decreased hyperalgesia during spontaneous heroin withdrawal. To gain information on the type of DR neuronal populations likely to play role in heroin withdrawal, we first determined the extent to which MOR mRNA is expressed in the four major classes of DR neurons, those that release only serotonin, GABA (VGaT neurons) or glutamate (VGluT3 neurons), as well as those that co-release glutamate and serotonin (VGluT3-TPH neurons). We found that less than 10% of DR neurons that release solely serotonin or glutamate, and less than 1% of neurons that co-release serotonin and GABA co-expressed MOR mRNA. In contrast, we established that most of the DR-neurons that expressed MOR mRNA either release only GABA (~37%) or co-release glutamate and serotonin (~27%). Thus, whereas these findings from naïve mice indicate that different subpopulations of DR neurons might mediate heroin effects, via its interactions with MOR receptors, we found that within the total population of DR-MOR neurons, the GABA-releasing, glutamate releasing, and glutamate-serotonin co-releasing neurons were the major neuronal subpopulations activated in hyperalgesia. We also demonstrated that inhibition of DR-GABAergic neurons blocked the expression of hyperalgesia during spontaneous heroin withdrawal. Collectively, we provided evidence indicating that activation of MOR-GABAergic, MOR-glutamate releasing and MOR-glutamatergic-serotonergic neurons, with non-overlapping DR distribution, might play a role in spontaneous opioid withdrawal behaviors. Furthermore, we provide evidence that inhibition of DR-GABAergic neurons blocks hyperalgesia.

We established  the functional involvement of DR neurons in hyperalgesia in heroin dependence by demonstrating that non-specific neuronal inhibition of DR neurons decreased hyperalgesia during spontaneous heroin withdrawal in male and female heroin-dependent mice. These findings indicate that both inhibitory and excitatory DR neurons potentially have a role in the expression of hyperalgesia during spontaneous withdrawal in heroin dependence.

Pharmacological studies have implicated the DR-serotonergic system in opioid effects [10, 11] and associations between the DR-serotonergic and GABAergic systems have been suggested based on results from ex vivo electrophysiological studies [13]. However, it is unclear the extent to which these two systems play a role in different aspects of opioid withdrawal, and the possible cellular and molecular mechanisms that mediate DR-serotonergic and GABAergic participation in opioid withdrawal.

The expression of MOR within DR neurons has been well documented by several anatomical approaches including the neuronal detection of MOR mRNA in the rat by using in situ hybridization [20], and DR immunodetection of the reporter mCherry in MOR-mCherry knock-in mice [21]. We extended these observations by demonstrating that MOR mRNA is expressed in different subpopulations of DR neurons, including neurons that release solely serotonin, GABA or glutamate, as well as in neurons that co-release glutamate and serotonin. While several studies had implicated both DR-serotonin and DR-GABA neurons in mediating brain opioid effects, we demonstrated that MOR are also expressed in DR-VGluT3 and in DR-VGluT3-TPH neurons, indicating that activation of MOR in the DR is likely to regulate release of glutamate-alone and co-release of glutamate and serotonin. We found that within the total population of DR-MOR expressing neurons the major populations of MOR mRNA expressing neurons were GABAergic and dual glutamatergic-serotonergic. Thus, it remains to be determined the extent to which previous studies suggesting opioids-induced changes in the DR serotonin system were due to release of serotonin alone or due to co-release of serotonin with glutamate.

We have demonstrated that the expression of hyperalgesia during heroin withdrawal correlated with neuronal increases of c-Fos expression within the DR [9]. By general inhibition of DR neuronal activity, we now demonstrated that this inhibition resulted in decreased hyperalgesia during withdrawal in heroin-dependent mice. Towards gaining an understanding of the possible molecular and cellular mechanisms mediating DR hyperalgesia, we phenotyped DR neurons co-expressing c-Fos and found that within the total population of DR-c-Fos-MOR neurons, most of them release GABA or glutamate alone or co-release glutamate and serotonin. While DR serotonin neurons mediate some opioids effects [10, 11], we did not detect activation within the subpopulation of DR-MOR neurons that releases only serotonin. The lack of detection of c-Fos in DR-MOR neurons that release solely serotonin and the increase of c-Fos in DR-GABAergic neurons, may be explained in part by prior findings demonstrating that DR-GABAergic neurons inhibit neighboring DR-serotonergic neurons [27]. In addition, pharmacological studies reported low extracellular levels of 5-HT in the DR during opioid withdrawal [10, 11], and ex vivo electrophysiological recordings showed inhibition of DR-serotonergic neurons during morphine withdrawal [13]. Collectively, it seems that opioid withdrawal involves a molecular-cellular mechanism in which DR-MOR-GABAergic are activated and their local GABA release results in inhibition of serotonergic neurons with concomitant decreases in the local release of serotonin.

A prior ex vivo electrophysiological study reported a decreased inhibition of DR-GABAergic neurons during spontaneous withdrawal in morphine-treated male mice [28]. We extended these observations by demonstrating that in male and female mice, DR-GABAergic neurons expressing MOR were activated during spontaneous withdrawal, which were preferentially located in the lateral subregions of the rostral DR. We further demonstrated that chemogenetic inhibition of DR-GABAergic neurons blocked the expression of hyperalgesia during spontaneous heroin withdrawal. From these results, we concluded that DR-GABAergic neurons play a crucial role in heroin withdrawal.

In contrast to previous studies ascribing a role to DR-GABAergic and DR-serotonergic neurons in opioid functions, to our knowledge, the current study is the first to provide evidence indicating that two subpopulations of DR-glutamatergic neurons expressing MOR play a role in the neurobiology of opioids. These are DR-MOR neurons expressing VGluT3 alone or in combination with serotonergic markers. Within this context, we had previously demonstrated that indeed DR-VGluT3 neurons release glutamate [25], and those neurons co-expressing VGluT3 and TPH co-release glutamate and serotonin [26].

We demonstrated that DR neurons play a role in the expression of hyperalgesia during spontaneous heroin withdrawal and that hyperalgesia involves, in part, the activation of DR-MOR neurons that mediate diverse forms of neurotransmission. Furthermore, we demonstrated that DR-GABAergic neurons play a role in hyperalgesia during spontaneous heroin withdrawal.

## Supplementary information

Supplementary Information
