# Supplementary material for: Inhibition of dorsal raphe GABAergic neurons blocks hyperalgesia during heroin withdrawal
Source: Neuropsychopharmacology. 2023 Jun 3;48(9):1300–8. doi: 10.1038/s41386-023-01620-5 (PMC10354084; doi:10.1038/s41386-023-01620-5)
Supplement: Supplementary file 1 — Supplementary Information [file 41386_2023_1620_MOESM1_ESM.docx]

**Supplementary Information**

**Supplemental Methods and Material**

**Drugs**

We dissolved diamorphine hydrochloride (heroin), dispensed by the National Institute on Drug Abuse, Intramural Research Program Pharmacy (Baltimore, MD, USA), in sterile saline (0.9% sodium chloride; Hospira, IL, USA). To induce opioid dependence, we subcutaneously (s.c.) injected increasing doses of heroin (5, 10, 20, and 40 mg/kg) twice daily from day 2 to day 5 (Fig. 1A) as previously described[9]. We dissolved JHU37160 dihydrochloride (J60) in sterile saline and administered intraperitoneally (i.p.) at 1 mg/kg.

**Assessment of mechanical sensitivity**

The testing apparatus consisted of an elevated platform (92 cm length × 28 cm width × 71 cm height) with a stainless-steel mesh floor (0.4 cm × 0.4 cm) and rectangular transparent compartments (10 cm × 10 cm × 13 cm) on top. We assessed mechanical sensitivity by applying a single unbending filament perpendicularly to the mid-plantar surface of either the left or right hind paw. The force at which the mouse retracted its paw in response to the stimulation (paw withdrawal threshold in gram-force [gf]) was recorded automatically by the von Frey device. We measured the withdrawal thresholds in adjacent boxes until we assessed them for each mouse at least once. We repeated the process five times, alternating between the left and right hind paws for a total of six measurements for each mouse (three per paw).

**Surgery details**

The mice were anesthetized with isoflurane (4-5% for induction, 1-2% for maintenance) and placed in a stereotaxic frame. Their skull was exposed and leveled. Viral injections were performed at a flow rate of 70 nl/min using a UltraMicroPump with a Micro 4 controller, 5 μl syringe, and 33-gauge needle (Hamilton Company, NV, USA). The needle was left in place for an additional 3 min to prevent reflux. In all surgical procedures, the mice were given the anti-inflammatory meloxicam (2 mg/kg; s.c.) to minimize postsurgical pain and discomfort.

**Histological verification of DR injections**

After the chemogenetic inhibition experiments, all mice were deeply anesthetized with an injection of chloral hydrate (8 mg/kg, i.p.) and transcardially perfused with 1X phosphate buffer (PB) followed by 4% paraformaldehyde (PFA). We kept the brains in 4% PFA for 2 h and then in a 18% sucrose solution overnight at 4°C prior to freezing (-80°C). We collected coronal serial cryosections (30 μm thick) from the DR of each mouse with a cryostat (CM3050 S, Leica, DC, USA). Free-floating coronal sections were incubated overnight at 4°C with rabbit anti-TPH (1:1000) and mouse anti-mCherry (1:1000) in PB that was supplemented with 4% bovine serum albumin (BSA) and 0.3% Triton X-100. After rinsing three times (10 min each) with PB, the sections were incubated with donkey anti-rabbit Alexa Fluor-488 (1:100) and donkey anti-mouse Alexa Fluor-594 (1:100) secondary antibodies for 1 h at room temperature. The sections were mounted on slides. Fluorescent images were collected with an Olympus FV1000 Confocal System. Images were taken sequentially with different lasers at 5X (low) and 20X (high) magnification objectives.

**RNAscope in situ hybridization combined with immunohistochemistry**

For the identification of DR neurons expressing MOR mRNA, we anesthetized drug-naive mice with an injection of chloral hydrate (8 mg/kg, i.p.) and transcardially perfused them under RNAse free conditions with diethyl pyrocarbonate (DEPC)-treated PB followed by 4% PFA. We kept the brains in 4% PFA for 2 h and then in a 18% sucrose solution in overnight at 4°C prior to freezing (-80°C). We collected coronal serial cryosections (16 μm thick) from the DR. Sections were incubated for 2 h at 30°C with mouse anti-TPH (1:1000) DEPC-treated PB with 4% BSA, 0.3% Triton X-100, and RNasin (5 μl/1ml). After rinsing three times (10 min each) with DEPC-treated PB, the sections were incubated with donkey anti-mouse Alexa Fluor-750 (1:100) secondary antibody for 1 h at 30°C. The sections were rinsed with DEPC-treated PB, mounted on Fisher SuperFrost slides, and dried overnight at 60°C. *In situ* hybridization was performed using the RNAscope Multiplex Fluorescent v1 assay according to the manufacturer’s instructions. The sections were treated with heat and protease digestion followed by hybridization with a mixture containing target probes to mouse *Slc32a1* (VGaT), *Slc17a8* (VGluT3) and *Oprm1* mRNA. *Oprm1* was detected by Atto-550, and VGaT by Atto-647 and VGluT3 by Alexa Fluor-488.

RNAscope *in situ* hybridization sections were viewed, analyzed, and photographed with a Zeiss LSM880 confocal microscope equipped with Airyscan/CY7.5 (Zeiss, White Plains, NY). Images were taken sequentially with different lasers at 5X (low) and 20X (high) magnification objectives. Pictures were adjusted to match contrast and brightness using Photoshop software. Neurons were counted if the stained cell was at least 5 μm in diameter and co-localized with the nuclear stain DAPI. The number of mice (*n* = 3/group; 12 sections/mouse) that were analyzed was based on previous studies in our laboratory[24]. For the DR rostrocaudal distribution we used a total of 9 brain sections/mouse, each being representative of the anatomical location that cover the rostro-caudal extend of the DR (from bregma -4.24 mm to -5.20 mm).

| **Experimental Models: Organism/Strains** | **Source** | **Identifier** |
| --- | --- | --- |
| Mouse: C57BL/6J mice | The Jackson Laboratory | CAT # 5657312 |
| Mouse: Slc32a1 (VGaT-ires-Cre) | The Jackson Laboratory | Cat# JAX:016962 |
| **Virus Strains** | **Source** | **Identifier** |
| AAV2-hSyn-hM4Di-mCherry | Addgene, MA, USA | CAT # 50475 |
| AAV2-hSyn-DIO-hM4Di-mCherry | Addgene, MA, USA | CAT # 44362 |
| AAV2-hSyn-DIO-mCherry | Addgene, MA, USA | CAT # 50459 |
| **Chemicals** | **Source** | **Identifier** |
| JHU37160 dihydrochloride (J60) | Hello Bio,  NJ, USA | CAT # HB6261 |
| RNasin | Promega**,** WI, USA | CAT # N2115 |
| **Critical Commercial Assays** | **Source** | **Identifier** |
| RNAscope Multiplex Fluorescent v1 assay | Advanced Cell Diagnostics, CA, USA | CAT # 320851 |
| **Antibodies** | **Source** | **Identifier** |
| Rabbit anti-TPH | ABclonal, MA, USA | CAT # A7147 |
| Mouse anti-mCherry | Takara Bio, Shiga, Japan | CAT # 632543 |
| Donkey anti-rabbit Alexa Fluor-488 | Jackson ImmunoResearch, PA, USA | CAT # 711-545-152 |
| Donkey anti-mouse Alexa Fluor-594 | Jackson ImmunoResearch, PA, USA | CAT # 715-585-150 |
| Rabbit anti-phospho-c-Fos | Cell Signaling Technology, MA, USA | CAT # 5348 |
| Mouse anti-TPH | Millipore-Sigma, MO, USA | CAT # T0678 |
| Donkey anti-rabbit Alexa Fluor-647 | Jackson ImmunoResearch, PA, USA | CAT # 711-605-152 |
| Donkey anti-mouse Alexa Fluor-750 | Abcam, MA, USA | CAT # ab175738 |
| **Oligonucleotides** | **Source** | **Identifier** |
| RNAscope probe *Slc32a1* (VGaT) | Advanced Cell Diagnostics, CA, USA | CAT # 319191 |
| RNAscope probe *Slc17a8* (VGluT3) | Advanced Cell Diagnostics, CA, USA | CAT # 431261 |
| RNAscope probe *Oprm1* | Advanced Cell Diagnostics, CA, USA | CAT # 489311-C2 |
| **Software** | **Source** | **Identifier** |
| Adobe Photoshop | Adobe, CA, USA | RRID: SCR_014199 |

**Table S1. Key Resources Table**

**Supplemental Figures**

**
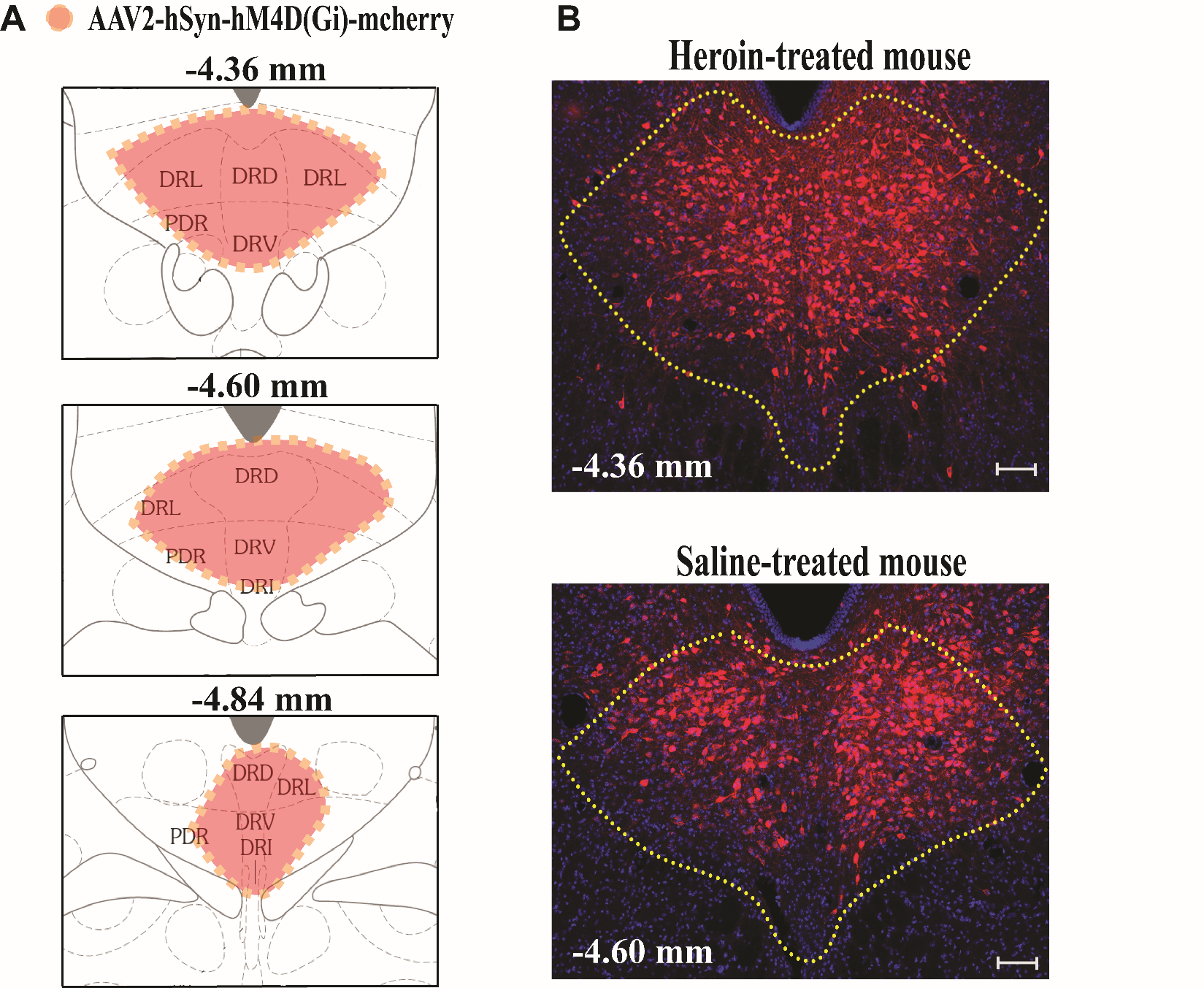
**

**Figure S1. Histological verification of viral expression in the DR. A.** Rostro-caudal extension of viral injections (AAV2-hSyn-hM4D (Gi)-mCherry) within the DR of heroin and saline-treated mice. **B.** Representative images (magnification of 5X) of the expression of AAV2-hSyn-hM4D (Gi)-mCherry in DR neurons at bregma -4.36 mm from a heroin-treated mouse and at bregma -4.60 mm from saline-treated mouse. Scale bars: 100 µm (C).

**
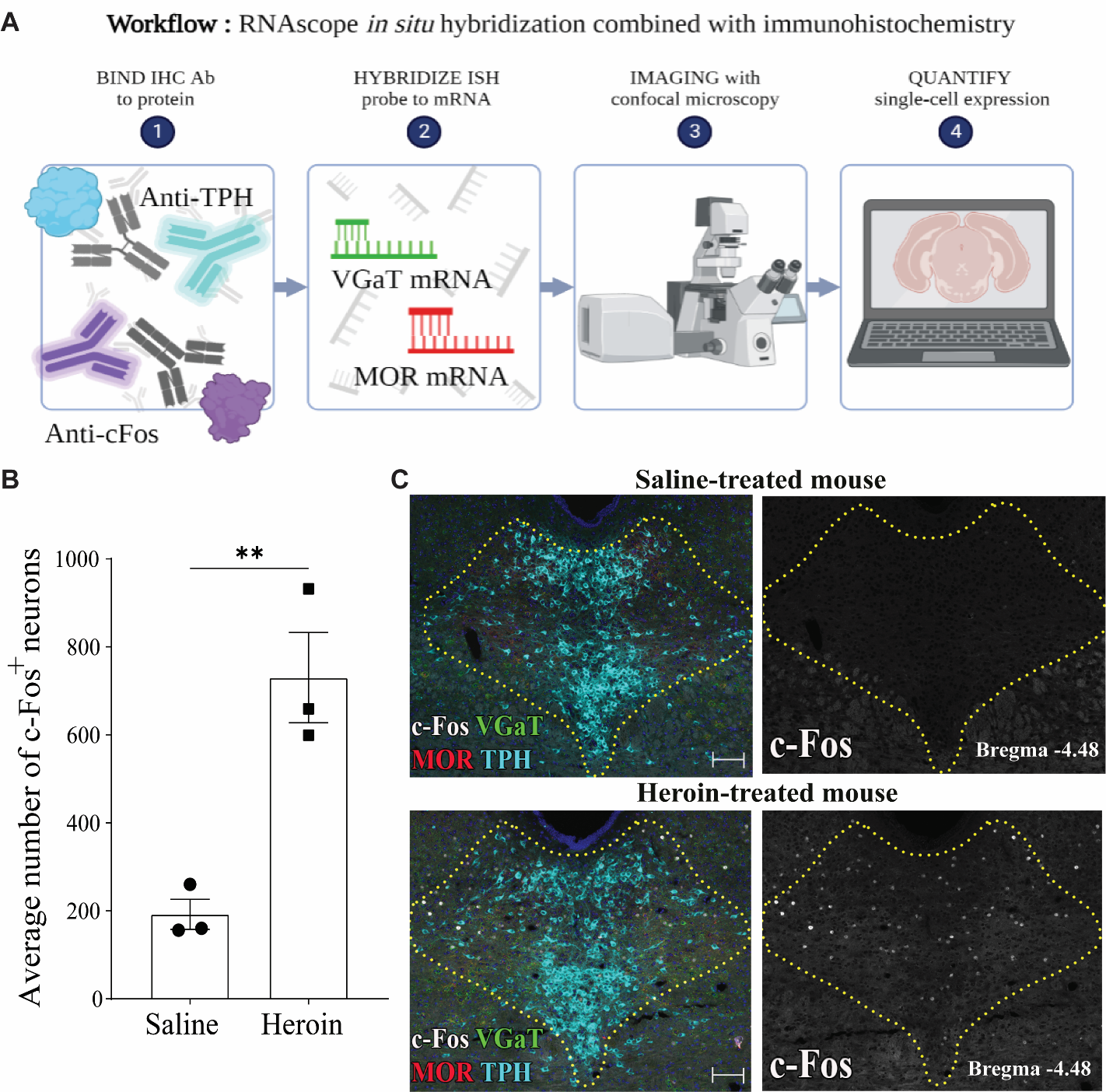
**

**Figure S2. Identification of DR-VGaT neurons expressing c-Fos in hyperalgesia during spontaneous heroin withdrawal.** **A.** Schematic representation showing the experimental procedure. **B.** Heroin-treated mice exhibited a higher number of c-Fos expressing neurons than saline-treated mice (***p* = 0.0038). **C.** Representative images (magnification of 5X) of the DR at bregma -4.48 from heroin- and saline-treated mouse. The data in **B** are expressed as mean ± SEM. (*n* = 3 mice/group). IHC, immunohistochemistry; ISH, *in situ* hybridization. Scale bars: 100 µm (C).

**
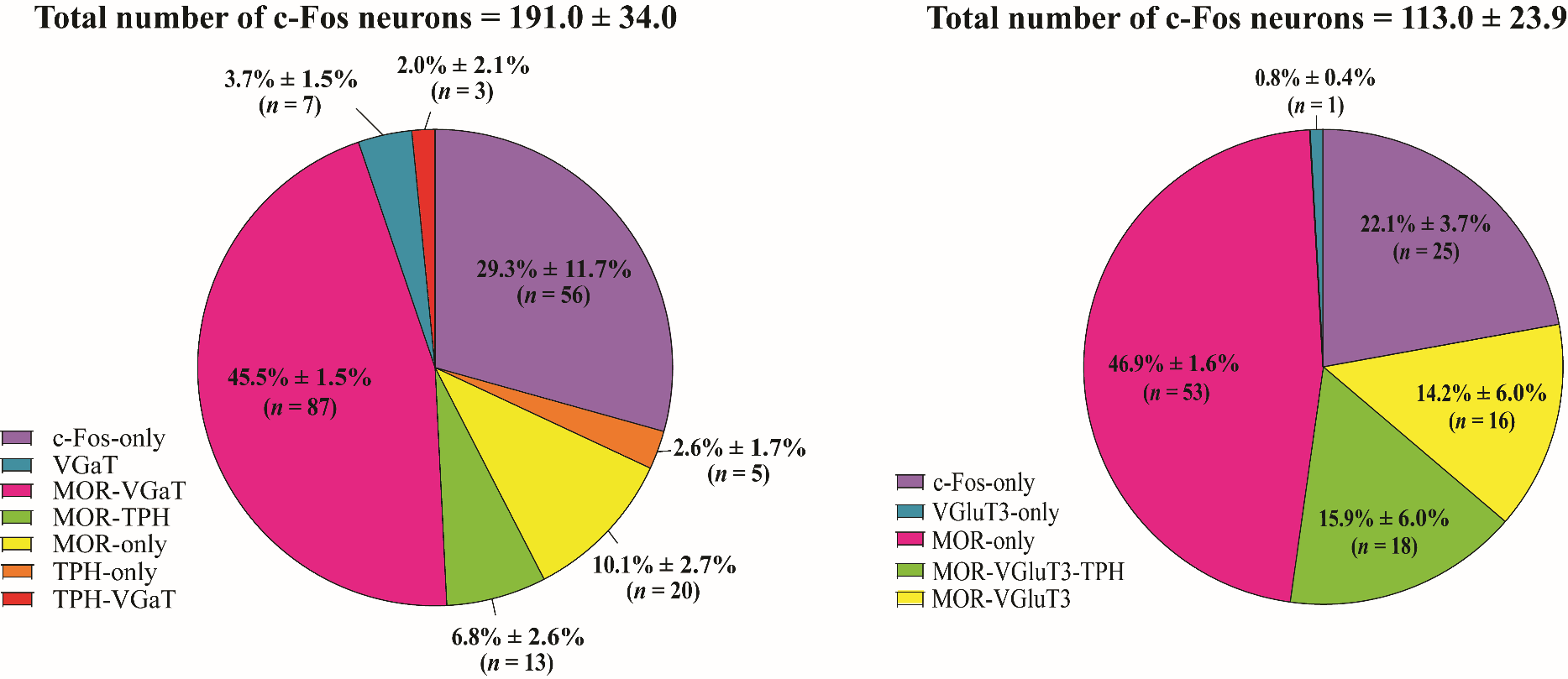
**

**Figure S3. Proportion of subpopulations of DR neurons expressing c-Fos in saline treated-mice.**

**
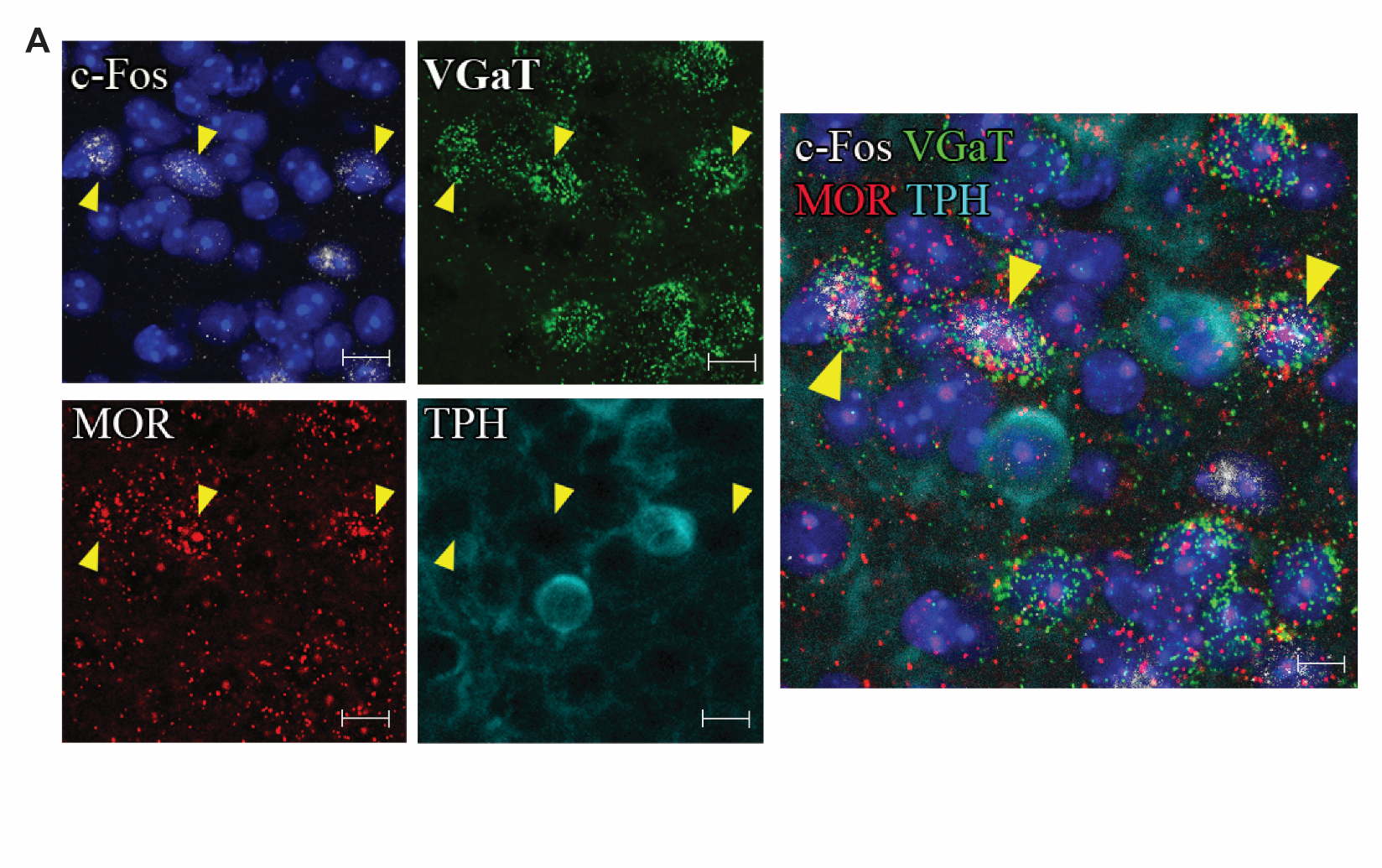
**

**Figure S4. DR expression of c-Fos-MOR-VGaT neurons.** Yellow arrowheads point to c-Fos-MOR-VGaT neurons at a magnification of 20X showing detection of c-Fos protein (white), VGaT mRNA (green), MOR mRNA (red), and TPH protein (cyan). Scale bars: 10 µm.


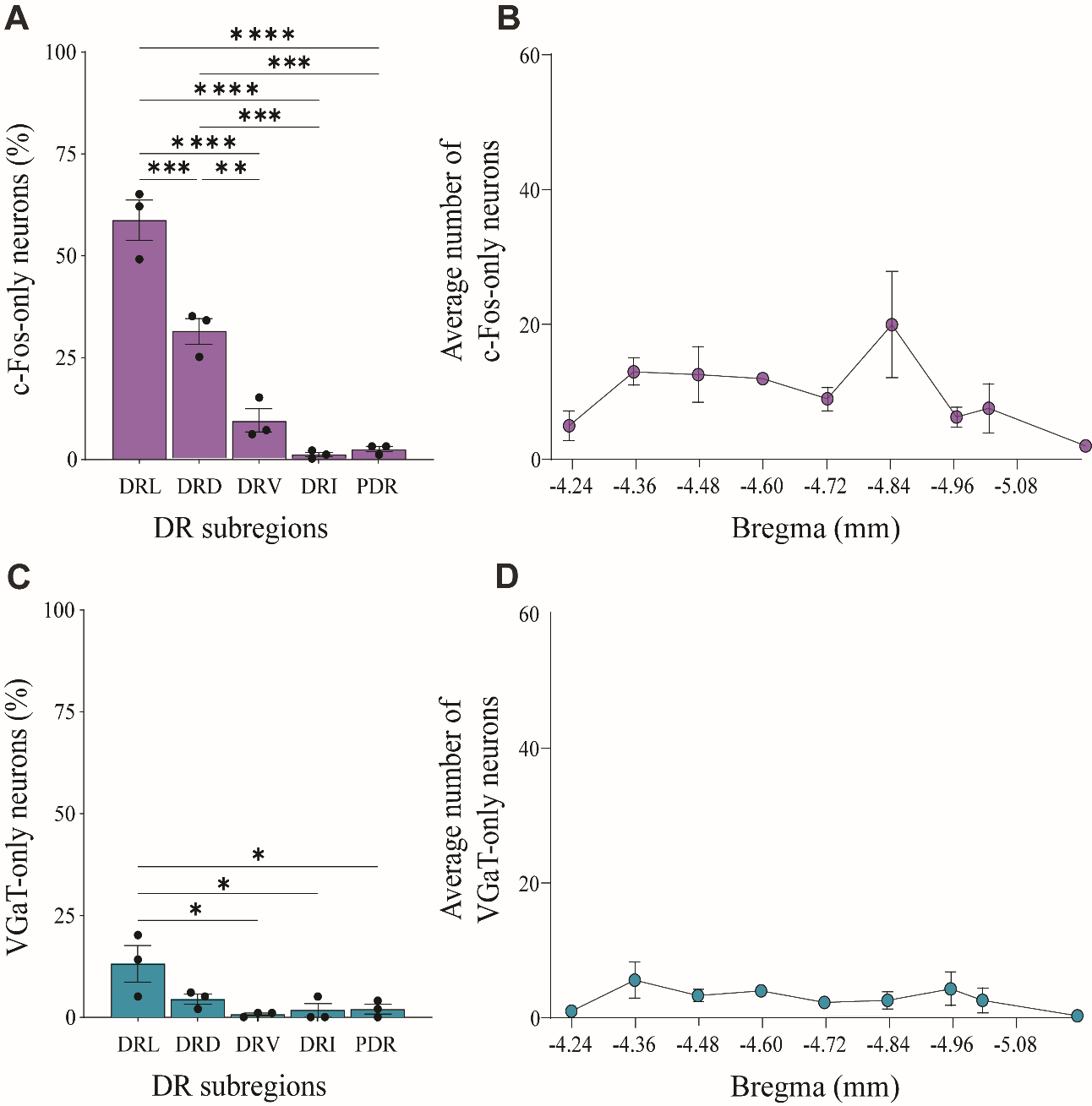


**Figure S5. Distribution of subpopulations in the DR of c-Fos neurons lacking the expression of MOR mRNA in hyperalgesia during spontaneous heroin withdrawal. A.** DR regional distribution of c-Fos neurons lacking the VGaT mRNA and MOR mRNA in heroin-treated mice showing higher concentration in DR lateral subregion (DRL) than DR dorsomedial (DRD; ****p* = 0.0005), DR ventromedial (DRV; *****p* < 0.0001), DR interfascicular (DRI; *****p* < 0.0001) and DR posterodorsal (PDR; *****p* < 0.0001) subregions. The c-Fos neurons lacking the VGaT mRNA and MOR mRNA were more concentrated in DRD subregions than in DRV (***p* = 0.0025), DRI (****p* = 0.0002) and PDR (****p* = 0.0003). **B.** DR rostrocaudal distribution of c-Fos neurons lacking the VGaT mRNA and MOR mRNA. **C.** DR regional distribution of c-Fos neurons expressing VGaT mRNA but lacking MOR mRNA in heroin-treated mice showing higher concentration in DRL subregion than DRV (**p* = 0.0187), DRI (**p* = 0.0306) and PDR (**p* = 0.0360) subregions. **D.** DR rostrocaudal distribution of c-Fos neurons expressing VGaT mRNA but lacking MOR mRNA. The data are expressed as mean ± SEM.


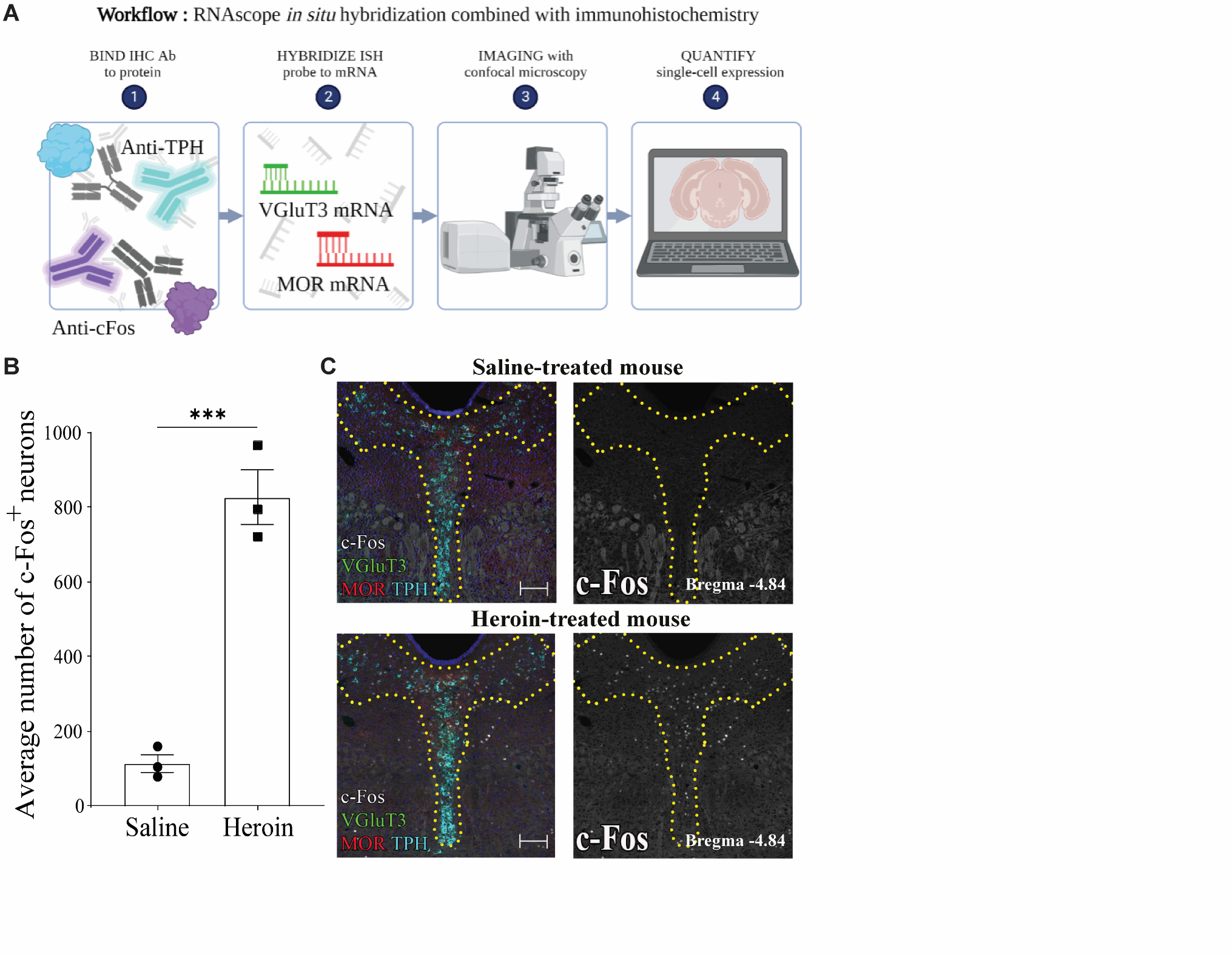


**Figure S6. Identification of DR-VGluT3 neurons expressing c-Fos in hyperalgesia during spontaneous heroin withdrawal.** **A.** Schematic representation showing the experimental procedure. **B.** Heroin-treated mice exhibited a higher number of c-Fos expressing neurons than saline-treated mice (****p* = 0.0004). **C.** Representative images (magnification of 5X) of the DR at bregma -4.84 from heroin- and saline-treated mice. The data in **B** are expressed as mean ± SEM. (*n* = 3 mice/group). IHC, immunohistochemistry; ISH, *in situ* hybridization. Scale bars: 100 µm (C).

**
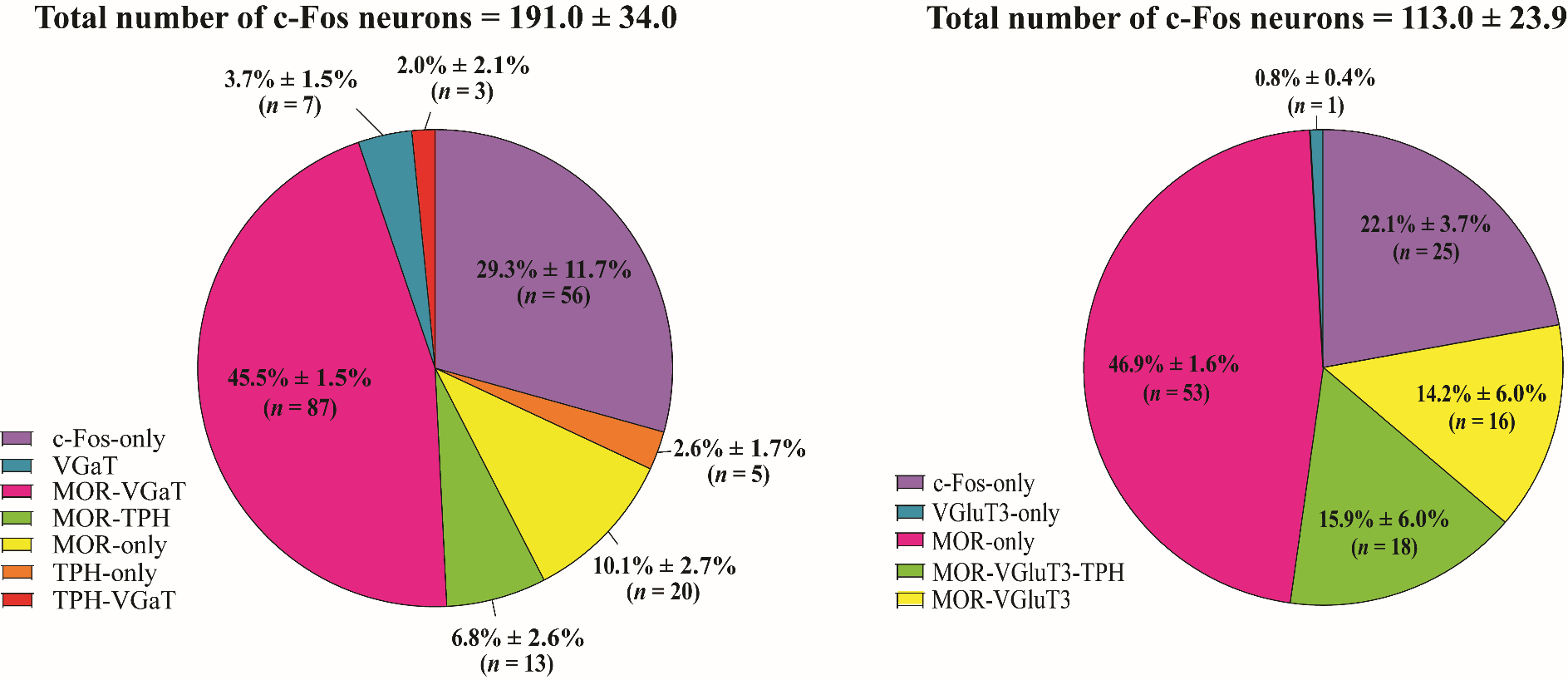
**

**Figure S7. Proportion of subpopulations of DR neurons expressing c-Fos in saline treated-mice.**

**
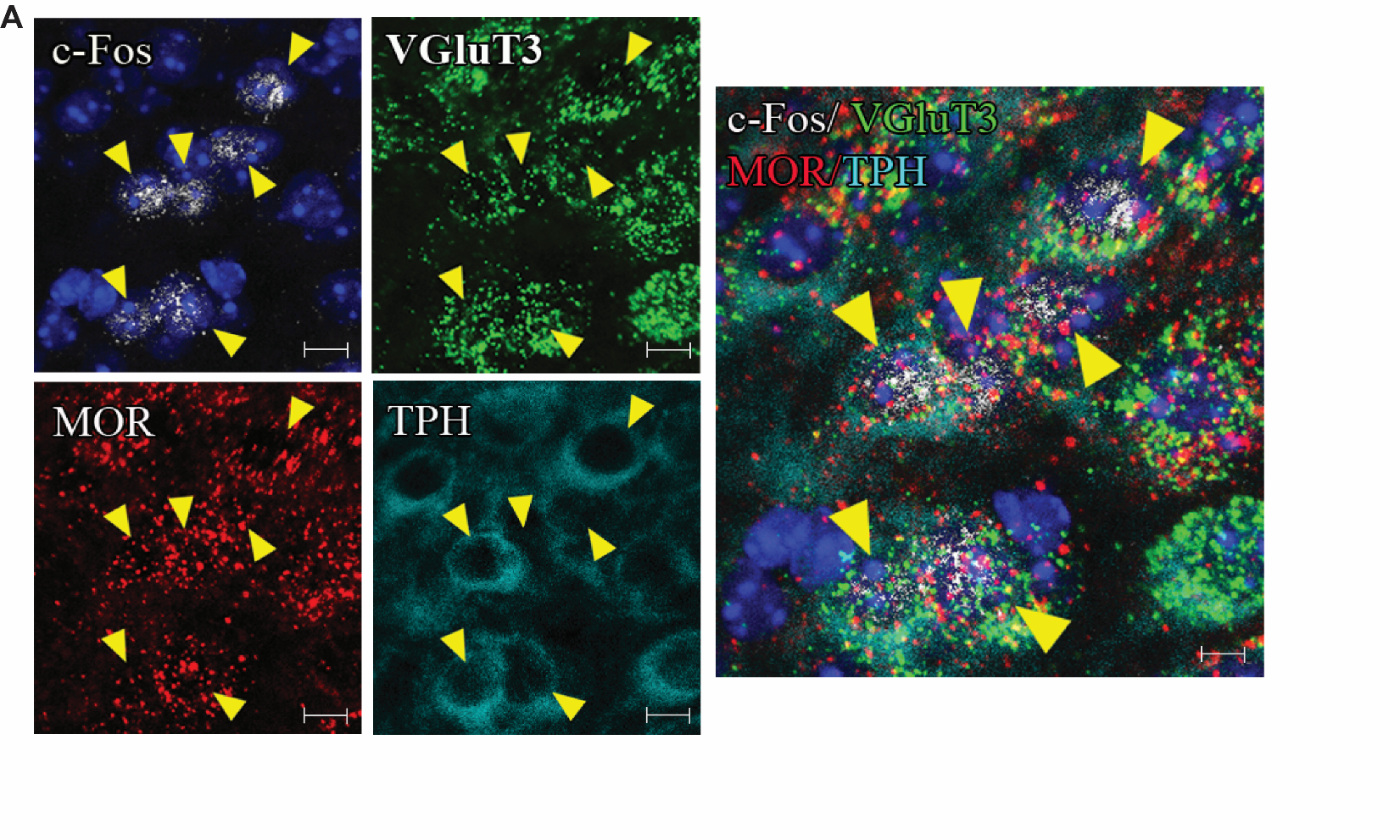
**

**Figure S8. DR expression of c-Fos-MOR-VGluT3-TPH neurons.** Yellow arrowheads point to c-Fos-MOR-VGluT3-TPH neurons at a magnification of 20X showing detection of c-Fos protein (white), VGluT3 mRNA (green), MOR mRNA (red), and TPH protein (cyan). Scale bars: 10 µm.


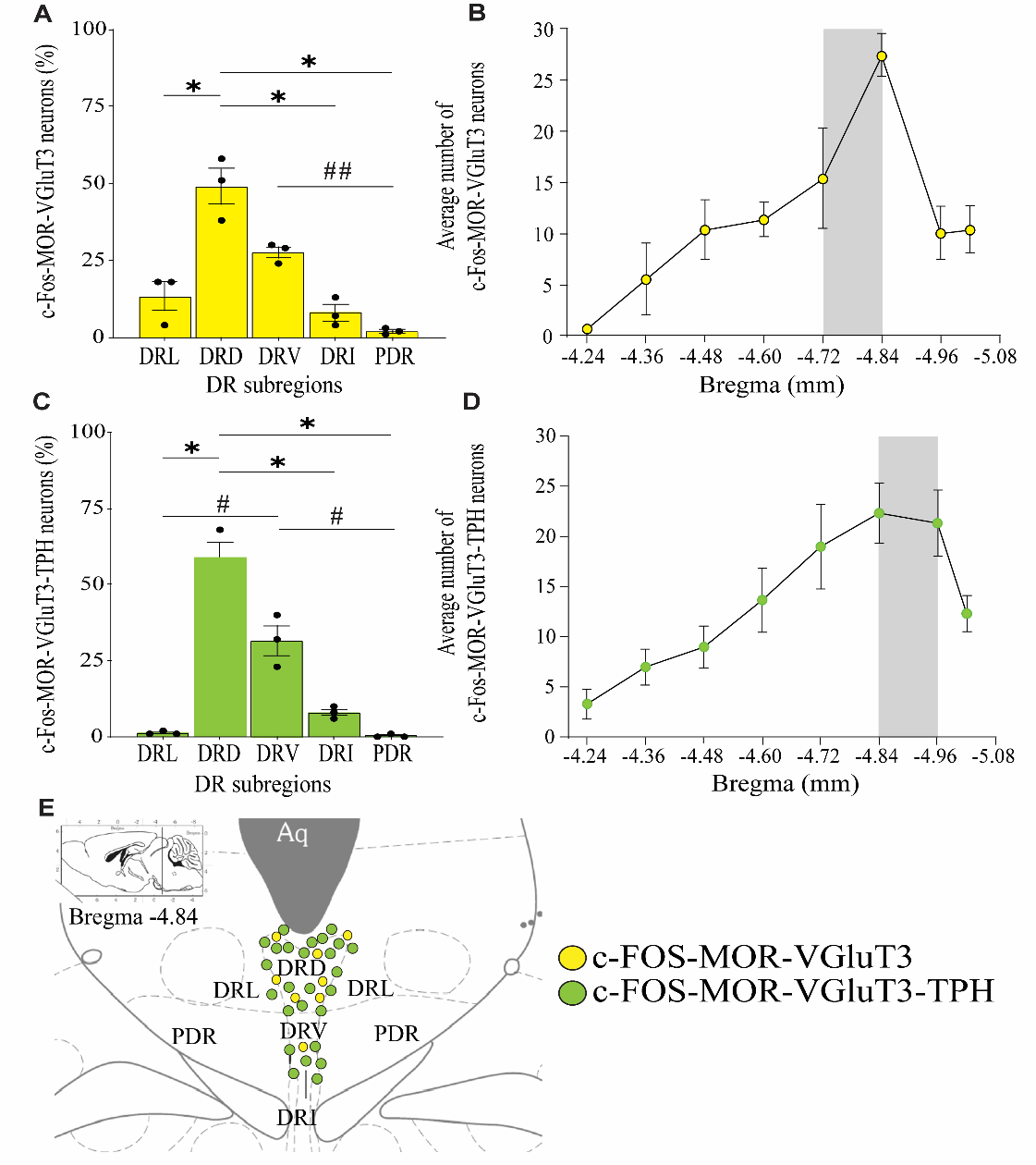


**Figure S9. DR distribution of c-Fos-MOR-VGluT3 and c-Fos-MOR-VGluT3-TPH neurons in heroin treated mice.** **A.** DR regional distribution of c-Fos-MOR-VGluT3 neurons in heroin treated mice showing higher concentration in the DR dorsomedial (DRD) subregion than in the DR lateral (DRL; **p* = 0.0367), DR interfascicular (DRI; **p* = 0.0163), or DR posterodorsal (PDR; **p* = 0.0105) subregions; and in the DR ventromedial (DRV) subregion than in the PDR (^##^*p* = 0.0087) subregion. **B.** DR rostrocaudal distribution of c-Fos-MOR-VGluT3 neurons. **C.** DR regional distribution of c-Fos-MOR-VGluT3-TPH neurons in heroin treated mice showing higher concentration in the DRD subregion than in the DRL (**p* = 0.0074), DRI (**p* = 0.0077), and PDR (**p* = 0.0066) subregions; and in the DRV subregion than in the DRL (^#^*p* = 0.0225) and PDR (^#^*p* = 0.0237) subregions. **D.** DR rostrocaudal distribution of c-Fos-MOR-VGluT3-TPH neurons. **E.** DR schematic representation showing concentration of c-Fos-MOR-VGluT3 and c-Fos-MOR-VGluT3-TPH neurons in the DRD and DRV subregions of the DR at bregma -4.84. The data in **A**-**D** are expressed as mean ± SEM. (*n* = 3 mice/group). Aq, aqueduct.


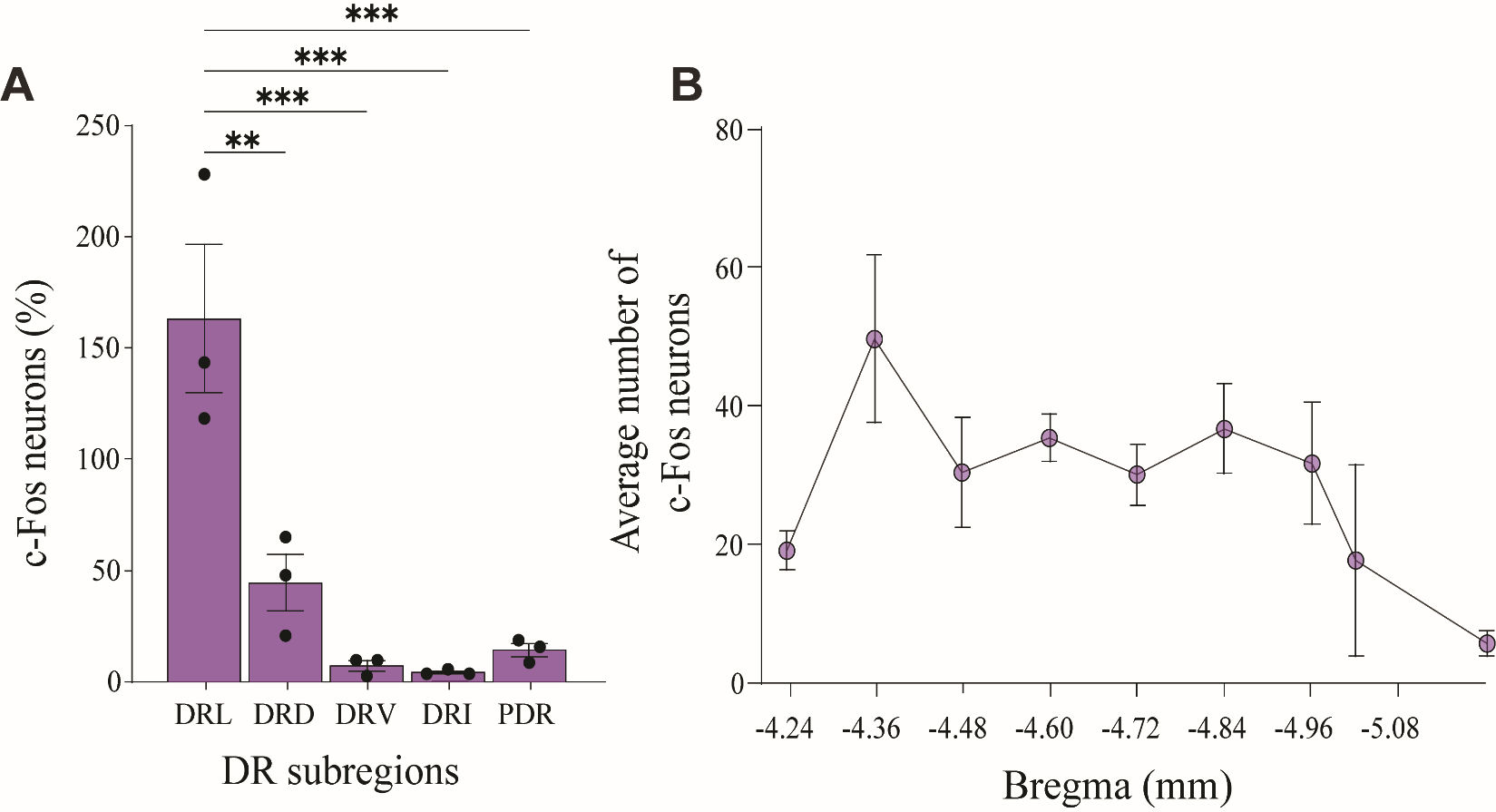


**Figure S10. Distribution of subpopulations in the DR of c-Fos neurons lacking MOR mRNA in hyperalgesia during spontaneous heroin withdrawal.** **A.** DR regional distribution of c-Fos neurons lacking MOR mRNA in heroin-treated mice showing higher concentration in DR lateral subregion (DRL) than DR dorsomedial (DRD; ***p* = 0.0013), DR ventromedial (DRV; ****p* < 0.0002), DR interfascicular (DRI; ****p* < 0.0001) and DR posterodorsal (PDR; ****p* < 0.0002) subregions. **B.** DR rostrocaudal distribution of c-Fos neurons lacking MOR mRNA. The data are expressed as mean ± SEM.
